# Cartilage Repair In Vivo: The Role of Migratory Progenitor Cells

**DOI:** 10.1007/s11926-014-0461-4

**Published:** 2014-09-21

**Authors:** Boris Schminke, Nicolai Miosge

**Affiliations:** Tissue Regeneration Work Group, Department of Prosthodontics, Georg August University, Robert-Koch-Str. 40, 37075 Göttingen, Germany

**Keywords:** Articular cartilage, Meniscus, Osteoarthritis, Rheumatoid arthritis, Migratory progenitor cells

## Abstract

The most common diseases of the joints and its tissues are osteoarthritis and rheumatoid arthritis, with osteoarthritis being anticipated to be the fourth leading cause of disability by the year 2020. To date, no truly causal therapies are available, and this has promoted tissue engineering attempts mainly involving mesenchymal stem cells. The goal of all tissue repairs would be to restore a fully functional tissue, here a hyaline articular cartilage. The hyaline cartilage is the most affected in osteoarthritis, where altered cell–matrix interactions gradually destroy tissue integrity. In rheumatoid arthritis, the inflammatory aspect is more important, and the cartilage tissue is destroyed by the invasion of tumor-like pannus tissue arising from the inflamed synovia. Furthermore, the fibrocartilage of the meniscus is clearly involved in the initiation of osteoarthritis, especially after trauma. Recent investigations have highlighted the role of migratory progenitor cells found in diseased tissues in situ. In osteoarthritis and rheumatoid arthritis, these chondrogenic progenitor cells are involved in regeneration efforts that are largely unsuccessful in diseased cartilage tissue. However, these progenitor cells are interesting targets for a cell-based regenerative therapy for joint diseases.

## Introduction

Mesenchymal stem cells (MSCs) reside in differentiated tissues and are often responsible for self-repair after trauma or disease. Their potential for a regenerative therapy of diseased cartilage has been recently summarized [1, 2]. MSCs are involved in the regeneration of mesenchymal tissues, for example, bone, cartilage, muscle, ligament, tendon, adipose, and stroma [3, 4, 5••, 6, 7], Pittenger et al. (1999) were the first to isolate adult MSCs from bone marrow and demonstrated their multilineage potential [8]. Subsequently, researchers isolated MSCs from various other adult tissues, such as blood, adipose, skin, mandible trabecular bone, muscle, synovial membrane and synovial fluid [9]. The diversity of the chondrogenic potential of MSCs isolated from these different tissues is still being investigated. An increased number of MSCs are observed in the synovial fluid of osteoarthritis (OA) patients [8]. Wakitani et al. performed the first transplantation of bone marrow-derived mesenchymal stem cells for cartilage repair in human OA, and a sort of cartilage-like tissue was observed after 42 weeks [10]. However, no single method is yet fully efficient for cartilage tissue regeneration by generating a functional tissue that lasts over time. In OA of late disease stages, the cartilage defects are often rather large, this, together with the cartilage-degrading milieu, makes tissue regeneration attempts especially difficult. MSCs have also been investigated in the context of rheumatoid arthritis (RA), here especially their immune modulatory properties are important [11].

OA is a degenerative joint disease with progressive loss of the articular cartilage and eburnation of the subchondral bone [12, 13••]. Furthermore, the disease is characterized as an whole organ disease [12, 13••] also involving the synovia [14, 15••] and the meniscus [16]. Recently, it has been demonstrated that the inflammation mediators found in OA are produced by the synovium, and not the cartilage tissue itself [17]. According to Reginster [18], nearly 1.75 million patients in England and Wales alone suffer from symptomatic OA, suggesting that it is the most common musculoskeletal disease worldwide. There is a strong association between increasing age and OA prevalence. Up to 20 % of the population over 60 years old shows signs of the disease [19]. OA often remains asymptomatic until late in the disease progression, and reliable early markers for diagnosis are still lacking. Therefore, total knee replacement is the gold standard treatment [19].

Meniscal lesions also lead to OA [16], and a high interdependency of OA with meniscal lesions has been described [20]. In total, 1.5 million knee arthroscopies are performed annually, and meniscal injuries account for more than 50 % of those operations [21, 22]. The prevalence of meniscal tears increases with age [23••] and may be as high as 56 % in men aged 70–90 years old [21]. Allografts or bio-engineered meniscal substitutes [24] can be applied after the removal of the meniscus; however, radiological and MRI scans show no protective effect against the development of OA [25]. The reasons for this lack of protection are still unknown. The failure to successfully remodel the allograft into a living tissue is one likely factor [26]. Therefore, almost all patients eventually require joint replacement [22].

Rheumatoid arthritis (RA) is the most common chronic inflammatory joint disease. It leads to progressive cartilage and bone destruction, but the time course and cellular mechanisms are somewhat different from those in OA. Full or partial work incapacitation will burden 35 % of RA patients within 10 years of RA onset [27]. Novel cell biological therapies have substantially improved patient outcomes [28]. However, the serious side effects of these biological therapies and the non-response rate of up to 40 or 50 % warrant the development of novel treatment options. Chronic synovial inflammation with tumor-like pannus overgrowth of the cartilage is responsible for the joint cartilage destruction. Fibroblast-like synoviocytes and synovial macrophages have been extensively investigated for their involvement in the inflammatory process through the production of proinflammatory cytokines, such as IL-1ß, IL-6, TNF-α, and matrix metalloproteinases [29]. Bone destruction in RA occurs mainly via the RANKL-dependent induction of osteoclasts [30]. Furthermore, CD4+ T-cells accumulate within the RA synovium [31, 32], and subpopulations mediate chronic synovial inflammation [33].

**Fig. 1:**
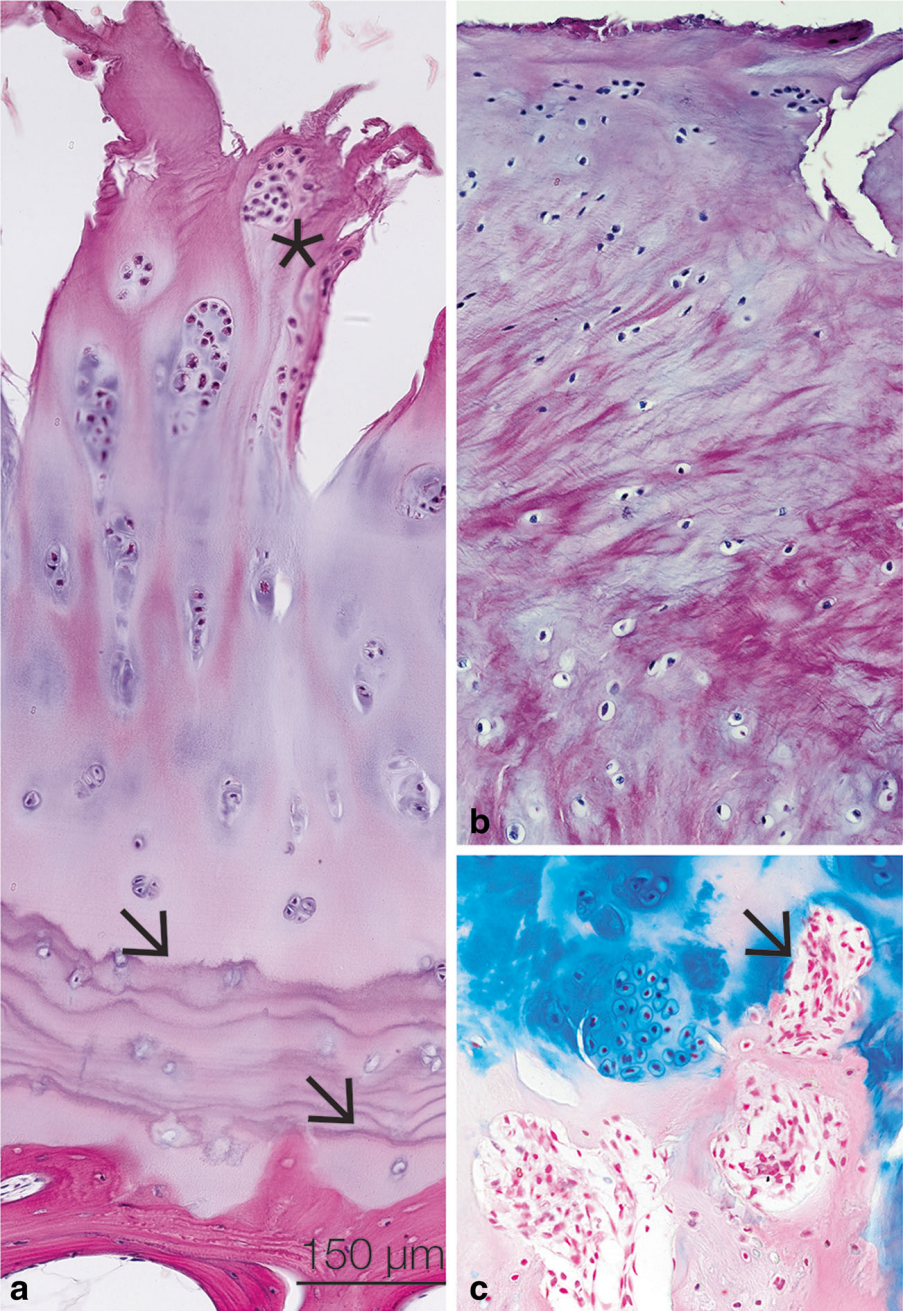
**a** Late stage OA from a patient after surgery for total knee replacement. Note the multiple tidemarks (*arrows*) and the cluster formation (*asterisk*). **b** Fibrocartilagenous repair tissue with scar-like appearance. **c** Mesenchymal tissue is entering the cartilage tissue (*arrow*) from underneath the tidemark allowing cell migration from the bone marrow into the diseased cartilage tissue. *Bar* (**a**–**c**) 150 μm

Once damaged or injured, the articular cartilage and the inner meniscus have limited intrinsic self-repair abilities (Fig. 1) because of their avascular nature. All regenerative therapeutic interventions need to address how to generate a repair tissue that has the same mechano-biological properties as the native hyaline articular cartilage. This neo-tissue also needs to integrate well with the articular cartilage in place [34]. To date, there is no method to derive a chondrogenic lineage from stem cells that will form functional hyaline cartilage tissue in vivo [35, 36]. Therefore, our approach is to utilize the repair cells present in the late stages of disease. In this review, we focus on the progenitor cells found in situ in osteoarthritic cartilage from OA patients, in the hyaline cartilage of RA patients, and in the inner, avascular part of the meniscus of OA patients. One line of research for cartilage repair is to optimize the performance of MSCs applied from outside, or to utilize the disease modulatory properties of these MSC. We introduce another concept, of investigating the role of chondrogenic progenitor cells found inside the diseased joint. To learn to understand their biology will render it possible to manipulate them in the future to utilize the potential of these repair cells already present in the diseased joint or to recruit progenitor cells to the diseased joint by enhancing their migration capacity.

### Migration of Stem Cells

**Fig. 2:**
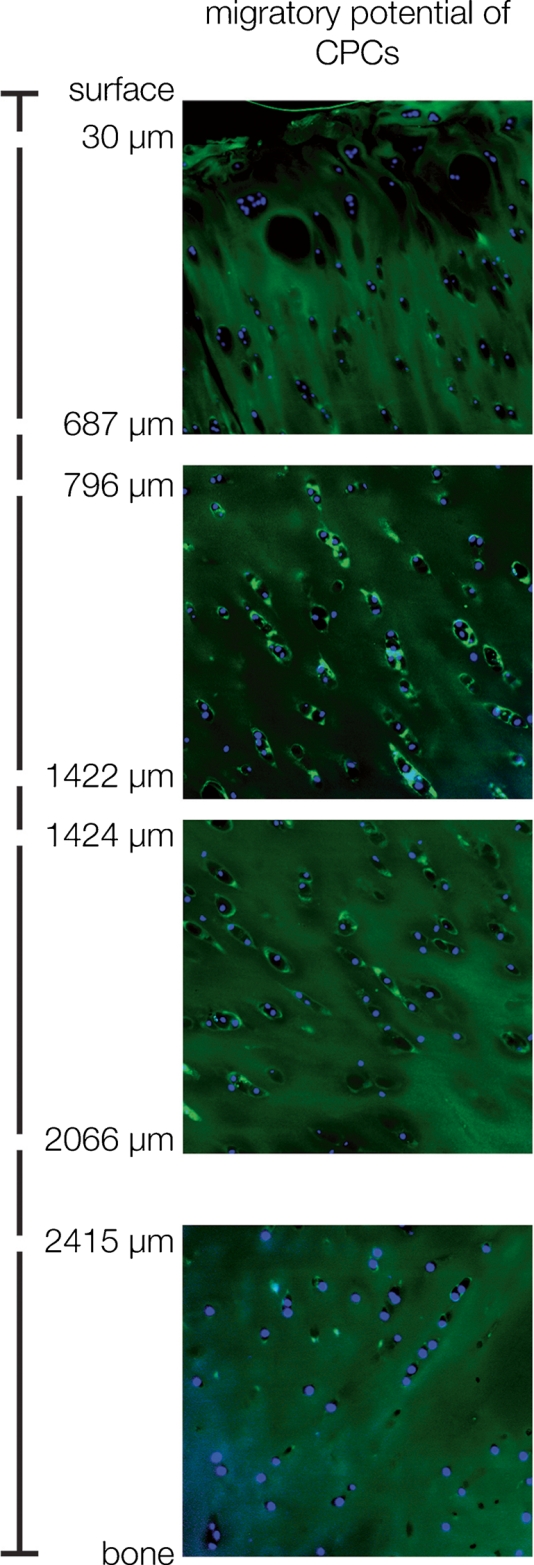
The migratory potential of chondrogenic progenitor cells from OA tissue in an ex vivo experiment. GFP-labeled cells were placed on OA tissue, and the cells migrated deep into the tissue. Taken from Koelling et al. [32] with permission of the publisher

The migration of epithelial and mesenchymal cells is a well-known mechanism that occurs during various biological processes [37]. Epithelial cell migration is essential for wound healing of the skin [38••], and mesenchymal cell migration is needed for many repair processes throughout life [39]. Hematopoiesis would not be possible without cell migration [40], and bone regeneration involves circulating osteogenic cells [41]. Furthermore, the basic biological processes of the stem cell niche, especially the generation of the transient, amplifying pool of progenitor cells, are dependent on migration processes [42]. Therefore, migratory capacity is a characteristic needed for proper differentiation of mesenchymal stem cells, although it is not listed in the current definition of mesenchymal stem cell characteristics, which include multi-differentiation potential, stem cell marker positivity, and clonicity. In this context, it is interesting to see that the progenitor cell populations from patient tissues from the late stages of OA exhibit a strong migratory potential at least in vitro (Fig. 2).

### Chondrogenic Progenitor Cells from OA Patients (CPCs)

**Fig. 3:**
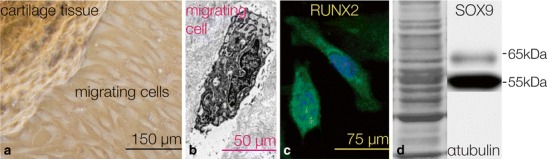
**a** Chondrogenic progenitor cells migrating from an OA cartilage sample to the surface of a culture dish. **b** A migrating cell at the ultrastructural level near the tidemark of OA tissue (used with permission from Koelling [32]). **c** The migratory chondrogenic progenitor cells are positive for Runx2 as shown by immunofluorescence (*green* staining), and **d** they are also positive for Sox9 as shown by western blotting

During the more advanced stages of OA, the fibrocartilaginous cartilage contains collagens, such as type I and type III [43–45], while we find the amount of collagen type II decreases as seen by quantitative immunohistochemistry [44]. In contrast, microarray investigations demonstrate an overall increased anabolism and an up-regulation of mRNAs also of cartilage-specific collagens [46, 47]. In any case, the altered matrix composition seems to be one reason for the long-term failure of the repair tissue to initiate *a restitutio ad integrim*. We identified chondrogenic progenitor cells (CPCs) as a subpopulation of cells found in the repair tissue of late stages of OA [48]. These cells not only exhibit a high migratory potential in vitro as well as ex vivo (Fig. 3) but they are positive for stem cell markers (for example, CD29, 73, 90, 105) and can be differentiated into adipocytes, cells of the osteoblastic lineage, and chondrocytes. The CPCs, also referred to as osteo-chondro progenitor cells [49], are under the control of the transcription factors Runx2 and Sox9 [48, 50]. RNA interference was applied to down-regulate the osteogenic transcription factor Runx2, with concomitant up-regulation of Sox9, the chondrogenic transcription factor, and consequently enhanced COL2A1 mRNA in an ex vivo experiment. Currently, we elaborated a knock-down/pull-down experiment combined with a proteomic analysis to identify adaptor molecules involved in the up-regulation of Sox9. This method will lead to a drug development strategy targeting small, modifying molecules that can enhance chondrogenesis during the late stages of OA. This multifaceted disease involves many diverse tissues, such as the synovium, fibrous capsule, hyaline cartilage, subchondral bone, and the meniscus, and has many pathogenic factors, such as extracellular matrix-degrading enzymes and its inflammatory cytokines. Therefore, this disease will not be substantially influenced by only one biological therapy. We envision a combinatorial approach with anti-inflammatory, anti-matrix-degrading measures, and the utilization of intrinsic progenitor cells.

### Chondrogenic Progenitor Cells from RA Patients (RA-CPCs)

**Fig. 4:**
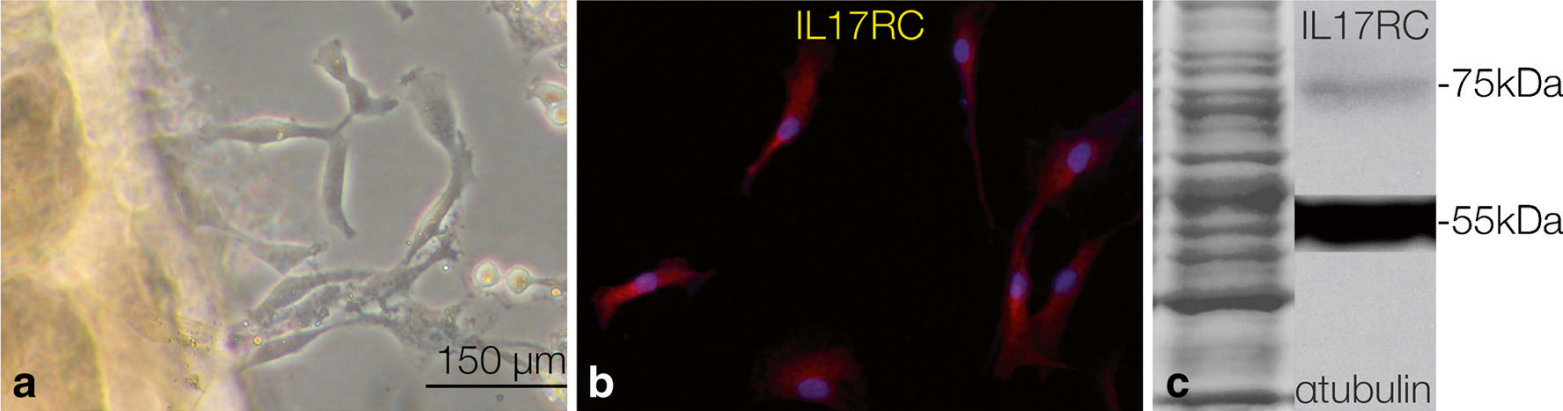
**a** Chondrogenic progenitor cells migrating from an RA cartilage sample. **b** The cells are positive for the IL17 receptor as shown here by immunocytochemistry and **c** by western blotting. *Bar* (**a**, **b**) 150 μm

Recently, we also characterized CPCs from diseased cartilage tissue from RA patients. These RA-CPCs are negatively influenced by interleukins present in the inflammatory environment of the RA joint and are therefore less chondrogenic (Fig. 4). RA-CPCs produce high levels of matrix metalloproteinases and proinflammatory cytokines under the influence of IL-17. Anti-inflammatory agents enable the cells to regain their chondrogenic capacity. Additionally, these RA-CPCs have high migration potential and can repopulate diseased cartilage tissue ex vivo. In vitro, IL-17A/F affects RA-CPC migration. In comparison, growth factors (EGF, IGF, PDGF) and the proinflammatory cytokine TNF-α, RA-CPCs can migrate equally well toward a gradient of EGF, TNF-α, or IL-17A/F (manuscript in preparation). This result underlines the important influence of inflammatory mediators on progenitor cells, especially on cell migration processes.

### Meniscus Progenitor Cells (MPCs)

**Fig. 5:**
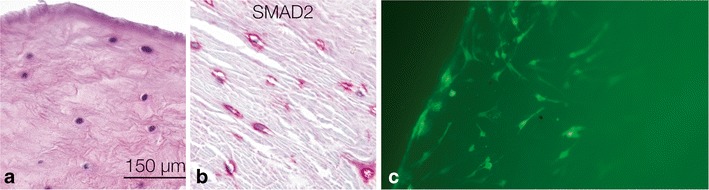
**a** The diseased meniscus is lacking the superficial zone with flattened cells and is only comprised of the round fibro-chondrocytes in the inner zone. **b** The meniscus progenitor cells (MPCs) are governed by the TGFβ pathway mediator Smad2, as shown here by immunohistochemistry. **c** Additionally, these MPCs migrate into diseased meniscus tissue. GFP-positive cells are found in the outer third of the OA meniscus. *Bar* (**a**–**c**) 150 μm

Over the last years, we have also investigated diseased human menisci from patients in the late stages of OA. A new classification system together with the results from transcriptomic and proteomic analyses enabled us to discriminate healthy and diseased human meniscus specimens. Interestingly, the samples receiving a high disease score also exhibited down-regulation of TGFß and Smad2. One consequence of the down-regulation of TGFß and Smad2 is the up-regulation of Runx2. The TGFß/BMP pathway, with its dual osteogenic and chondrogenic actions, is a good candidate for further investigation. We also identified progenitor cells in the inner, avascular part of the diseased human meniscus, similar to the CPCs and RA-CPCs found in the cartilage:. These meniscus progenitor cells (MPCs) normally produce collagen type I, and they are fibro-cartilagenous in nature [51]. MPCs also exhibit a high migratory potential (Fig. 5). The initial results indicate that MPCs are also governed by a balance between the transcription factors Runx2 and Sox9. The knock-down of Runx2 in MPCs enhances p-Smad2 and drives them towards the chondrogenic differentiation. In contrast, BMP2 stimulation of MPCs reduces Smad2 levels and enables the MPCs to move towards the osteogenic lineage [51].

### Chondrogenic Progenitor Cells and Their Role in Cartilage Repair

We have isolated migratory progenitor cell populations from late-stage human OA cartilage, RA cartilage, and osteoarthritic meniscus. These cells are positive for the well-known stem cell markers CD105, CD106, CD73, CD29 and Stro-1; however, their individual stem cell marker profiles vary. Whether the CPCs are unique from the cells exhibiting a similar stem cell marker profile in vitro, which have been identified in cartilage tissue after enzymatic digestion [52–55], has not been investigated, The relationship of CPCs to the cells found in the superficial zone of healthy cartilage tissue in vivo, also exhibiting a stem cell marker, is also not clear [56]. However, the CPCs differ in that they exhibit a high migratory potential [57], and all CPCs identified so far seem to be regulated by a balance between the transcription factors Runx2 and Sox9. The knock-down of Runx2 consistently enhances the chondrogenic potential of CPCs, RA-CPCs, and MPCs via by up-regulation of Sox9 and collagen II expression [48, 50, 51].

**Fig. 6:**
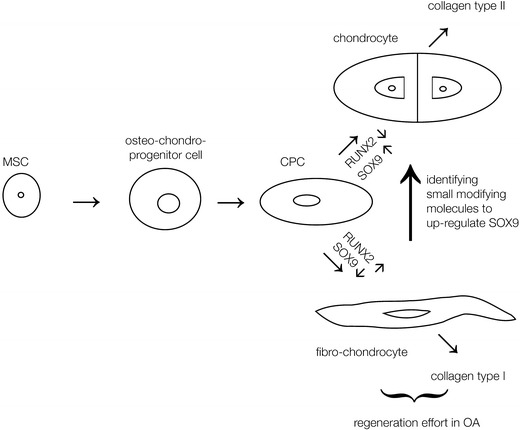
Schematic of our concept of chondrogenic progenitor cells in situ and their role in cartilage repair. Mesenchymal stem cells (*MSCs*) enter the cartilage tissue most likely from the bone marrow to become an osteo-chondro progenitor cell population. These osteo-chondro progenitor cells differentiate into chondrogenic progenitor cells (*CPCs*). In late stages of OA, they generate fibrocartilage rich in collagen type I in a scar-like repair tissue. Our aim is to drive these CPCs in situ into the chondrogenic lineage by the manipulation of the transcription factors Runx2 and Sox9

Our studies on progenitor cells have revealed exciting data with a future potential for the clinical application of CPCs for cartilage repair [48, 50, 51]. Our aim is to manipulate these progenitor cells in situ with the help of small modifying molecules to enhance their chondrogenic potential. We suggest that the resident cells in the diseased cartilage tissue are the ideal candidates for in situ manipulation for regenerative therapy applications (Fig. 6). These cells are already active in the diseased tissue and may be more efficient and safer than exogenous cells. To date, we have elucidated the chondrogenic pathways of progenitor cells to find adaptor molecules of two of their master regulators, Sox9 and Runx2, to promote Sox9 expression. However, even if we succeed in identifying small molecules that enhance Sox9 expression (and thereby the synthesis of collagen type II), further research is required. Obviously, the strong influences of age, gender, and body weight on the regenerative capacity of the progenitor cells should be considered, as has already been described for MSCs [58]. Further, the guidance of these cells to the diseased area has to take into account the surrounding tissue, which is filled with negative mediators that promote degradation and inflammation. Other groups have also investigated the migratory potential of CPCs, and have found that interleukin-1 beta and tumor necrosis factor alpha inhibit their migration [59]. In line with this, CPCs also exhibit a tendency to over-express chemokines that promote chemotaxis possibly involving their general migratory potential [60].

## Conclusion

In recent years, progress has been made in understanding the pathogenesis of OA and human articular cartilage degrading processes. The involvement of migratory progenitor cells has become more important. However, many details of the biological mechanisms governing these cells remain to be elucidated. How do we tip the balance of the transcription factors Runx2 and Sox9 to favor the latter to enhance chondrogenesis in the progenitor cells? Additionally, how do we use their migratory capacity to guide them toward the lesion? Taken together, we suggest that the manipulation of migratory progenitor cells in situ might be a feasible way to facilitate the regeneration efforts in vivo to enhance the *restitution ad integrim* in osteoarthritic cartilage tissue.
